# Rasch analysis of the Neck Bournemouth Questionnaire: Turkish version, validity, and reliability study

**DOI:** 10.3906/sag-1907-3

**Published:** 2019-12-16

**Authors:** Yasemin ÖZEL ASLIYÜCE, Derya GÖKMEN, Özlem ÜLGER

**Affiliations:** 1 Faculty of Physiotherapy and Rehabilitation, Hacettepe University, Ankara Turkey; 2 Department of Biostatistics, Faculty of Medicine, Ankara University, Ankara Turkey

**Keywords:** Health-related quality of life, disability, chronic neck pain

## Abstract

**Background/aim:**

The multidimensional evaluation of patients with chronic neck pain is important for planning the treatment program. The aim of this study was to investigate the validity and reliability of the Turkish version of the Neck Bournemouth Questionnaire (NBQ).

**Materials and methods:**

The internal construct validity of the NBQ was examined by the fit of the data to the Rasch measurement model. External validity of the NBQ was evaluated by testing for expected associations of Rasch transformed NBQ score with the corresponding variables through the process of convergent validity. The reliability of the NBQ in terms of both internal consistency and test-retest reliability was assessed by the person separation index (PSI) and differential item functioning (DIF) by time effect.

**Results:**

It was determined that the questionnaire has 2 factors. None of the items of Factor 1 (F1) and Factor 2 (F2) showed DIF. The reliability of F1 (Cronbach’s alpha = 0.89, PSI = 0.87) and F2 (Cronbach’s alpha = 0.77, PSI = 0.87) was good with Cronbach’s alpha and PSI. There was a good correlation between NBQ/F1 and the Neck Disability Index (NDI) (r = 0.673) and Neck Pain and Disability Scale (NPDS) (r = 0.709). Also, there was a correlation between NBQ/F2 and the Beck Depression Inventory (BDI) (r = 0.552) and Beck Anxiety Inventory (BAI) (r = 0.410).

**Conclusion:**

The Turkish version of the Neck Bournemouth Questionnaire is valid and reliable.

## 1. Introduction

Neck pain is a health problem that nearly half of all individuals in the world experience at least once in their lifetime [1]. It is known that physical, behavioral, and mental health is adversely affected, the level of disability increases, and the level of health-related quality of life decreases significantly in patients with neck pain [2,3]. The appropriate use of outcome measurements is very important to determine the most effective treatment program depending on the evaluation. While measurement questionnaires designed to assess pain, disability, and quality of life for patients with low back pain are relatively common, they are limited for patients with neck pain. For these reasons, the Neck Bournemouth Questionnaire (NBQ) was adapted by Bolton and Humphreys in 2002 from the Bournemouth Questionnaire, which was developed for low back pain [4]. The Turkish version of the Bournemouth Questionnaire, which was developed for patients with low back pain, has been shown to be valid and reliable [5]. 

The NBQ consists of 7 questions that examine the pain intensity, daily life activities, social activities, anxiety, emotional aspects of depression, kinesiophobia, and the ability to control pain. The items in the questionnaire are specific to patients with neck pain and each question evaluates a different parameter. In this context, the Neck Pain and Disability Scale (NPDS) [6], Neck Disability Index (NDI) [7], Beck Depression Inventory (BDI) [8], Beck Anxiety Inventory (BAI) [9], and Tampa Scale for Kinesiophobia Scale (TSK) [10] were used to evaluate the validity of the questionnaire. Because the items of the questionnaire are short and clear, it provides practicality for researchers and clinicians during the application. The NDI [11] and NPDS [12] are the most frequently used questionnaires for patients with neck pain. The advantages of the NBQ over the NDI and NPDS are the evaluation of anxiety, depression, and kinesiophobia, as well as being shorter, more practical, and more sensitive in measuring the time-dependent change of symptoms [13,14]. There are 5 versions of this questionnaire (Italian, German, Portuguese, Dutch, and French) but there is no Turkish version available [13,15–18]. Therefore, the aim of this study is to investigate the validity and reliability of the Turkish version of the NBQ.

## 2. Materials and methods

The study was approved by the Hacettepe University Noninvasive Clinical Research Ethics Committee (Ethics Committee Registration No: GO 17/844). Written permission was obtained from Jennifer E. Bolton on 18.09.2017 for the Turkish version of the NBQ formed by translation and cultural adaptation.  One hundred and twenty-five patients (18–65 years old) with neck pain (at least for the previous 3 months) due to mechanical or cervical disc herniation were included in the study. The participants signed a consent form to be included in the study. Individuals who were illiterate, with malignant disease, with motor weakness due to the herniated cervical disc, or with loss of function due to disease were excluded from the study. For the test-retest study, there were 1–3 days between the first and second administration of the NBQ. For the test-retest reliability, 43 individuals were planned to be included, but during this time, the patients who had a change in the severity of their symptoms due to additional treatment were excluded and the study was completed with 40 individuals. 

The translation and cultural adaptation of the NBQ were carried out according to the guidelines established by Beaton et al., as follows:

1st step-Translation: The translation of the NBQ was carried out by a physiotherapist and a linguist whose native language is Turkish and can speak English fluently as well. The two people who created the translation independently created two separate translation texts.

2nd step-Synthesis: The two translators discussed each version and created a consensus version.

3rd step-Back Translation: The questionnaire was translated back to English by two linguists whose native language was Turkish and could speak Turkish fluently.

4th step-Expert Committee Review: Five physiotherapists with at least 2 years of experience in the field of low back and neck health and 2 native English speakers created an expert committee. The physiotherapists included in the committee have performed studies of validity and reliability in this field. The expert committee evaluated the translations in terms of cultural adaptation and conformity, and formed the prefinal version of the questionnaire.

5th step-Pretesting: The last version of the questionnaire was applied as a pilot to 35 patients and it was determined by the expert committee that the questionnaire was understandable.

For the external validity of the NBQ, questionnaires that are known to be valid and reliable in patients with neck pain were used. The NDI was developed to assess the level of disability in patients with neck pain. The NDI consists of 10 questions in total. The subsections of the NDI are designed to assess pain, personal care, lifting, reading, headaches, concentration, work, driving, sleeping, and recreation [7]. The NPDS assesses factors such as pain severity, participation in social life, sleep, mood, driving, and stiffness in the neck. The NPDS consists of 20 questions in total [6]. The BDI was prepared to measure the individuals’ behaviors and thoughts specific to depression. In the BAI, the questions assess the tendency to anxiety. The BAI and BDI consist of 21 questions that are scored between 0 and 3 [8,9]. The TSK includes injury/reinjury and fear-avoidance parameters in work-related activities. The TSK consists of 17 questions [10].

### 2.1. Statistical analysis

#### 2.1.1. Validity

Factor analysis was performed to assess the unidimensionality of the NBQ prior to continue with Rasch modeling. The internal construct validity of the NBQ was examined by the fit of the data to the Rasch measurement model [19], while the external validity of the NBQ was assessed by testing for expected associations of Rasch transformed NBQ score with the corresponding variables through the process of convergent validity. The Rasch analysis includes the sequential steps [20] of (i) rescoring of NBQ items showing disordered thresholds; (ii) after deletion of the misfit items, analysis for overall model and individual item fit; (iii) examination for differential item functioning (DIF) for sex, age (≤44/>44), body mass index (BMI ≤26/>26), and duration of pain (DP ≤48/>48); and (iv) test for local independency and unidimensionality. In terms of external validity, the association of Rasch transformed NBQ score with the NDI, NPDS, BDI, BAI, and TSK was analyzed by Spearman’s correlation coefficient.

#### 2.1.2. Reliability

The reliability of the NBQ in terms of both internal consistency and test-retest reliability was examined by the person separation index (PSI) and DIF by time effect. The PSI [21], which is equivalent to Cronbach’s alpha [22] but has the linear transformation from the Rasch model, is a measure of internal consistency. Minimum Cronbach’s alpha values of 0.7 and 0.90 are suggested for group and individual use, respectively. For test-retest reliability of the NBQ, DIF was carried out to verify the invariance of item difficulty hierarchy across the first and the second assessment (DIF by time). Data were analyzed using RUMM2020 [23].

## 3. Results

A total of 125 chronic neck pain (CNP) patients, 102 females and 23 males, were included in the study. The sex-based distribution of the sociodemographic characteristics of all participants is shown in Table 1. Bartlett’s test was 420.929 and the KMO was 0.83. According to the KMO and Bartlett test results, the number of samples is sufficient for factor analysis and the sample of our study is also suitable for factor analysis. As a result of the factor analysis, two factors with eigenvalues of >1 explained 67.2% of the total variance. Considering the content of the questionnaire, it was decided to interpret it with two factors. When the rotated factor loads of the items were examined, Items 1, 2, 3, 6, and 7 were found to be included in Factor 1 (F1), while 4 and 5 were included in Factor 2 (F2). 

**Table 1:** Fit of NBQ/F1 items to Rasch model.

Items	Location	SE	Individual item fit residual	Chi-square test statistics	P
Item 1	–0.463	0.053	–0.333	0.322	0.571
Item 2	0.293	0.052	–0.505	0.674	0.412
Item 3	0.628	0.055	–1.246	3.475	0.062
Item 6	–0.132	0.050	0.841	0.025	0.874
Item 7	–0.326	0.052	1.664	1.128	0.288

SE: Standard error

Interpretability of the NBQ was assessed by the percentage of incomplete questionnaires and the percentage of respondents reporting the best or worst score (ceiling and floor effect). Since none of the respondents had incomplete data for all the items of the NBQ, the floor (ceiling) effects were 0 (0%), 0 (1.6%), and 4.8% (0.8%) for the total, F1 subscale, and F2 subscale, respectively.

### 3.1. Rasch analysis of NBQ/F1 (Items 1, 2, 3, 6, 7) 

Starting with 5 items, two items (Items 3 and 7) displayed disordered thresholds and thus the adjacent categories were collapsed together. Following this, all items were found to fit the model (given a Bonferroni adjustment fit level of 0.01) (Table 1). Overall mean item fit residual was 0.084 (standard deviation (SD) 1.158) and mean person fit residual was –0.451 (SD 1.193). Item-trait interaction was nonsignificant, supporting the invariance of items (chi-square: 5.62 (df = 5), P = 0.344). When DIF was tested for the variables mentioned above, none of the items showed DIF. The scale also satisfied the requirements of local independence and unidimensionality.

The PSI was good (0.89), indicating the ability of the scale to differentiate between 4 groups of patients, and Cronbach’s alpha was 0.87. When the test-retest reliability was examined via DIF by time, none of the items showed DIF.

**Figure 1:**
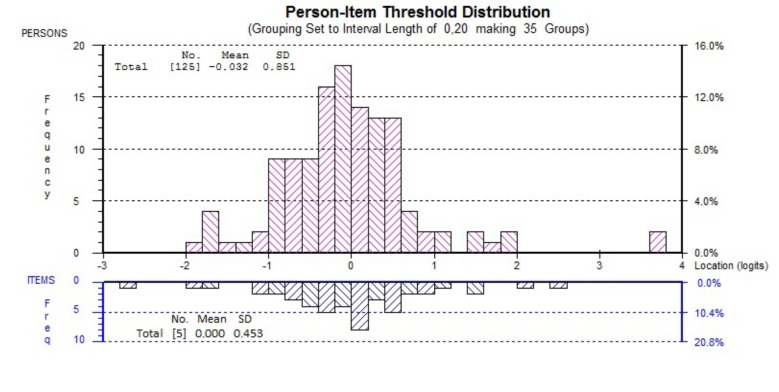
Targeting of NBQ/F1 to patients.

When the targeting of the final 5-item NBQ/F1 was evaluated, the scale was well-targeted to the patients with a mean person score of –0.032 and mean item score of 0 (Figure 1).

### 3.2. Rasch analysis of NBQ/F2 (Items 4, 5) 

Starting with 2 items, Item 4 displayed disordered thresholds, thus necessitating the classification of adjacent categories together. Following this, both items were found to fit the model (given a Bonferroni adjustment fit level of 0.025) (Table 2). Overall mean item fit residual was 0.172 (SD 0.404) and mean person fit residual was –0.626 (SD 0.879). Item-trait interaction was nonsignificant, supporting the invariance of items (chi-square: 2.29 (df = 2), P = 0.318). When DIF was tested for the variables mentioned above, none of the items showed DIF. The scale also satisfied the requirements of local independence and unidimensionality.

**Table 2:** Fit of NBQ/F2 items to Rasch model.

Items	Location	SE	Individual itemfit residual	Chi-square teststatistics	P
Item 4	–0.011	0.059	0.457	1.397	0.237
Item 5	0.011	0.054	–0.114	0.893	0.345

SE: Standard error

The PSI was good (0.77), indicating the ability of the scale to differentiate between 3 groups of patients, and Cronbach’s alpha was 0.78. When the test-retest reliability was examined via DIF by time, none of the items showed DIF.

**Figure 2:**
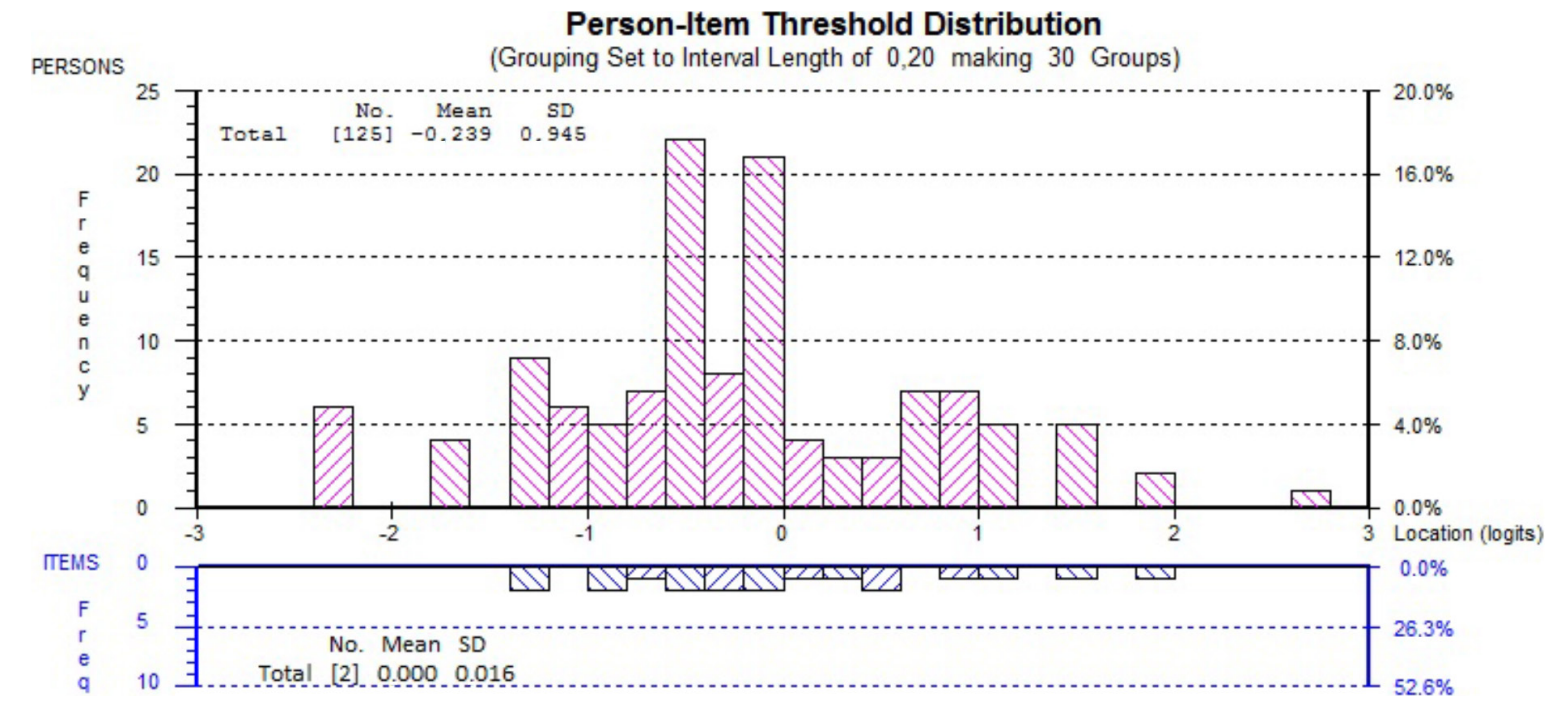
Targeting of NBQ/F2 to patients. SD: Standard deviation

When the targeting of the final 2-item NBQ/F2 was evaluated, patients on average had lower “disability” levels (mean person score: –0.239) than the average difficulty of the scale items (mean item score: 0) (Figure 2).

### 3.3. External construct validity

When the correlations of NBQ Rasch transformed scores with the NDI, NPDS, BDI, BAI, and TSK were examined, there was a positive correlation between NBQ/F1 and the NDI (r = 0.673), NPDS (r = 0.709), BDI (r = 0.338), BAI (r = 0.405), and TSK (r = 0.330). There was also a positive correlation between NBQ/F2 and the NDI (r = 0.359), NPDS (r = 0.458), BDI (r = 0.552), BAI (r = 0.410), and TSK (r = 0.223) (Table 3).

**Table 3:** Results of the external validity.

	NBQ/F1		NBQ/F2	
Variables	r	P	r	P
NDI	0.673	<0.001	0.359	<0.001
NPDS	0.709	<0.001	0.458	<0.001
BDI	0.338	<0.001	0.552	<0.001
BAI	0.405	<0.001	0.410	<0.001
TSK	0.330	<0.001	0.223	0.013

F1: Factor 1 F2: Factor 2 NBQ: Neck Bournemouth Questionnaire NDI: Neck Disability Index NPDS: Neck Pain and Disability Scale BDI: Beck Depression Inventory BAI: Beck Anxiety Inventory TSK: Tampa Scale for Kinesiophobia

## 4. Discussion

As a result of this study, it was determined that the Turkish version of the NBQ, a suitable biopsychosocial model developed for patients with CNP, is valid and reliable. According to the results of the factor analysis, it was found that the questionnaire had a two-factor structure. F1 included items related to pain and function, and F2 included items related to anxiety and depression.

Each item of the NBQ represents a different field. Each field represented can be affected by various parameters such as cultural characteristics, age, and pain duration. Since the content of the questionnaire is so rich, it is more appropriate to determine its validity and reliability by using a modern psychometric approach, Rasch analysis. The Rasch analysis allows the total score to be converted to the linear score. 

According to the factor analysis, it was determined that the two factors of the NBQ explain the total variance better, and when the content is examined, it was determined that the questionnaire has two factors. In the original article about the questionnaire published by Bolton et al. in 2002, the questionnaire was interpreted with a single-factor structure [4]. In the Italian version of the NBQ, published in 2014 by Geri et al., it was found that the questionnaire had a two-factor structure for the first time. Then, according to Rasch analysis of the Italian version of the NBQ, which was published by Geri et al. in 2015, it was determined that the NBQ had two factors. According to this study, items 1, 2, 3, 6, and 7 are included in F1 while items 4 and 5 are included in F2. F1 was defined as “pain and function” and F2 was defined as “anxiety and depression” [13,24]. Our results are consistent with the studies published in the literature in the recent years. When the factor loadings of each item in the questionnaire are taken into consideration, items 1, 2, 3, 6, and 7 are included in F1 and items 4 and 5 are included in F2. 

Items 3, 4, and 7 displayed disordered thresholds. When we examine the contents of these items, we think that they have important contributions to the NBQ and it is necessary for the protection of the biopsychosocial aspect of the question.

The internal consistency of the Turkish version of the NBQ was quite high (Cronbach’s alpha was 0.87). The PSI value (0.89) was also good. The high Cronbach’s alpha and PSI indicated that the variables in the study were homogeneous and the questionnaire was reproducible. 

In this study, according to personal differences such as sex, age, BMI, and duration of pain, no item showed DIF. This shows that the answers to the questions are not affected by these variables. In addition, the absence of time-dependent DIF also indicates that the test-retest reliability is high and the reliability does not change within a certain period of time. 

External construct validity is analyzed by valid and reliable scales and questionnaires for the individuals included in the study [25]. The NPDS, NDI, BDI, BAI, and TSK were used in our study and the highest correlation level was between the NPDS and NBQ. The reason for the strongest relationship between these two questionnaires is that both of the questionnaires are evaluating pain and function, as well as depression. However, although the content of the two questionnaires seems similar, the NBQ is richer in content in terms of anxiety and kinesiophobia [14]. The stronger relation of the NDI and NPDS with F1 of the NBQ indicates that these questionnaires assess pain and function rather than anxiety and depression. In addition, we think that the NBQ has a low-intermediate relationship with the TSK, BAI, and BDI and this is due to the fact that these questionnaires and scales are not specific to individuals having neck pain.

According to Deyo et al., the ideal questionnaire is a short and practical one that minimizes the burden of data collection and analysis [26]. Based on our results, we think that the NBQ is an ideal questionnaire because it is short and practical, and it contains clear questions. It is known that the severity of symptoms associated with neck pain changes over time. For this reason, examining the NBQ results according to the time period is very important in terms of reflecting the clinical changes. 

As a result of our study, we also think that the NBQ contains all the parameters needed to evaluate the quality of life so it can give an idea about the quality of life of patients with CNP.

In conclusion, Rasch analysis showed that the Turkish version of the NBQ is valid and reliable for patients with CNP. The NBQ is practical, comprehensible, and suitable for a biopsychosocial model. It is sensitive to time-dependent changes and it is a questionnaire that provides objective results for the planning and maintenance of clinical trials as well.

## Disclaimers/Conflict of interest

The authors declare no conflict of interest. No funding has been received for this study.

## Informed consent

The study was approved by the Hacettepe University Noninvasive Clinical Research Ethics Committee (Ethics Committee Registration No: GO 17/844). All participants provided informed consent in the format required by the relevant authorities and/or boards.
